# Resurgence of Yellow Fever in Angola, 2015–2016

**DOI:** 10.3201/eid2210.160818

**Published:** 2016-10

**Authors:** Antoinette A. Grobbelaar, Jacqueline Weyer, Naazneen Moolla, Petrus Jansen van Vuren, Francisco Moises, Janusz T. Paweska

**Affiliations:** National Institute for Communicable Diseases of the National Health Laboratory Service, Johannesburg, South Africa (A.A. Grobbelaar, J. Weyer, N. Moolla, P. Jansen van Vuren, J.T. Paweska);; National Public Health Institute, Luanda, Angola (F. Moises)

**Keywords:** Yellow fever, outbreak, Angola, viruses, vector-borne infections

**To the Editor:** Yellow fever virus (YFV) is endemic in tropical and subtropical Africa and South America, and it is transmitted to humans and nonhuman primates through the bites of infected mosquitoes. The virus, a member of the family *Flaviviridae*, causes yellow fever, which in severe cases manifests as fulminant hemorrhagic fever. Outbreaks of yellow fever in humans occur mostly in the urban cycle of the virus, which involves its transmission through the bites of the day-feeding infected *Aedes aegypti* mosquitoes (*1*). As many as 130,000 cases with fever and jaundice or hemorrhage may occur annually with a concomitant 78,000 deaths (*2*). A low capacity for yellow fever diagnosis and lack of surveillance in disease-endemic countries likely contribute to case underreporting (*1*).

Although wide-scale yellow fever vaccination, which began in the 1940s and continued through 1960, resulted in a dramatic decrease in the number of outbreaks, waning population immunity and lapse of continued high coverage vaccination in yellow fever–endemic countries have led to the disease’s resurgence in Africa (*1**–**3*). Between 1980 and 2012, the World Health Organization received reports of 150 outbreaks in 26 countries in Africa (*2*). In the past 5 years (2011–2016), outbreaks have been documented in Democratic Republic of Congo, Sudan, Cameroon, Chad, Senegal, Côte d’Ivoire, Uganda, and Sierra Leone (*3*). During 2005–2016, Sudan was the most affected country; 3 outbreaks were reported, resulting in 1,508 cases and 368 deaths (*3**,**4*).

Yellow fever was first recognized in Angola in the 1930s, but not until 1971 (65 cases) and 1988 (37 cases) were sizeable outbreaks reported (*5**–**7*). As of July 1, 2016, a total of 3,552 suspected cases, including 875 laboratory-confirmed cases and 355 deaths, had been reported from all 18 provinces of Angola, with most cases occurring in Luanda Province (*8*). In this account, we provide laboratory confirmation that the first suspected viral hemorrhagic fever cases in Angola were YFV infections and report preliminary sequencing data.

On January 14, 2016, we received whole blood samples from 3 patients who resided in Luanda, Angola, and were suspected of having viral hemorrhagic fever. All were men, two 22 and one 30 years of age. Clinical manifestations in all patients were fever, headache, nausea, and vomiting. Myalgia, malaise, reduced consciousness, and jaundice each occurred in 2 patients; abdominal pain, back pain, ecchymosis, conjunctivitis, and bleeding gums each occurred in 1 patient. Two of the patients died 7 days after disease onset (Technical Appendix Table). Laboratory diagnosis consisted of testing for filoviruses, arenaviruses, and bunyaviruses, as well as for chikungunya and dengue viruses by using reverse transcription PCR (RT-PCR). All results of RT-PCRs were negative. A real time RT-PCR for YFV, targeting the 5′ noncoding region (*9*), produced positive results for samples from all 3 patients. The samples were then tested by using a pan-flavivirus RT-PCR targeting the flavivirus NS5 gene region using primers FU1 8993F and cFD2 9258R (*10*). Resulting amplicons of expected size (266 bp) obtained from 2 of the 3 samples were subjected to conventional Sanger sequencing. Phylogenetic analysis was performed by using a maximum-likehood method in MEGA version 6 (http://www.megasoftware.net) based on the general time reversible model under 1,000 bootstrap iterations, and sequence divergence was determined to calculate the p-distances between sequences. Phylogenetic inference of the sequence data demonstrated 95% nucleotide sequence similarity between the virus from this outbreak and the 14F YFV strain isolated in Angola in 1971 (Figure). PCR and sequencing results were reported to Angolan Public Health Institute on January 19, 2016.

**Figure:**
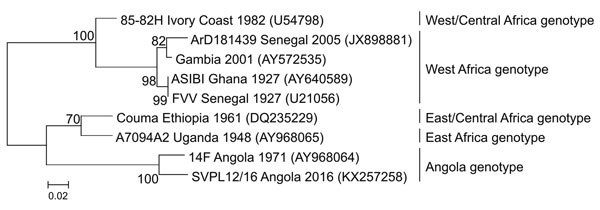
Maximum-likelihood phylogenetic reconstruction of 201 nt of the NS5 gene of yellow fever virus in Angola and other recognized genotypes of the virus in Africa. Node values indicate bootstrap confidence values generated from 1,000 replicates. GenBank accession numbers are indicated in brackets. Scale bar indicates substitutions per site.

The identification of the outbreak prompted cordon vaccination in Luanda in February 2016, followed by mass vaccination in other areas (*8*). The initially localized outbreak in Angola developed into the biggest and most widespread yellow fever epidemic recorded in Africa for decades (*3**,**8*). Sequencing and phylogenetic analysis indicate that the outbreak virus is highly similar to that identified during the epidemic in Angola in 1971. This finding reiterates the endemicity of yellow fever in Angola and emphasizes the need for consistent routine mass vaccination of the at-risk population to prevent future outbreaks.

Technical Appendix Demographic and clinical characteristics of the first 3 case-patients with confirmed yellow fever, Angola, 2015–2016.
