# Supplementary material for: Resurgence of Yellow Fever in Angola, 2015–2016
Source: Emerg Infect Dis. 2016 Oct;22(10):1854–5. doi: 10.3201/eid2210.160818 (PMC5038398; doi:10.3201/eid2210.160818)
Supplement: Technical Appendix — Demographic and clinical characteristics of the first 3 case-patients with confirmed yellow fever, Angola, 2015–2016. [file 16-0818-Techapp-s1.pdf]

Article DOI: http://dx.doi.org/10.3201/eid2210.160818

Resurgence of Yellow Fever in Angola, 2015–2016

Technical Appendix

Technical Appendix Table. Demographic and clinical characteristics for first 3 confirmed yellow fever case-patients, Angola, 2015–2016

Characteristic	Case-patient 1	Case-patient 2	Case-patient 3
Age/sex	22 M	22 M	30 M
Date of yellow fever onset	.2015 Dec 25	.2015 Dec 5	2015 Dec 7
Date of death	.2016 Jan 1	2015 Dec 12	–
Fever	Yes	Yes	Yes
Headache	Yes	Yes	Yes
Nausea/vomiting	Yes	Yes	Yes
Myalgia	No	Yes	Yes
Arthralgia	–*	–	Yes
Abdominal pain	–	Yes	–
Back pain	No	Yes	–
Malaise	Yes	Yes	–
Reduced consciousness	Yes	Yes	–
Ecchymosis/purpura	Yes	No	–
Conjuctivitis	Yes	No	–
Bleeding gums	Yes	No	–
Jaundice	Yes	Yes	–

* –, no data provided.
